# Examining the Use and Application of the WHO Integrated People-Centred Health Services Framework in Research Globally – a Systematic Scoping Review

**DOI:** 10.5334/ijic.7754

**Published:** 2024-04-25

**Authors:** Osama Hafiz, Xuejun Yin, Shiying Sun, Jingsong Yang, Hueiming Liu

**Affiliations:** 1 Kings College London, United Kingdom; 2 School of Population Medicine and Public Health, Chinese Academy of Medical Sciences & Peking Union Medical College, Beijing, China; 3 The George Institute for Global Health, University of New South Wales, Sydney, Australia; 4 Menzies Centre for Health Policy and Economics, The University of Sydney, Sydney, Australia; 5 Sydney Institute for Women, Children and Families, Sydney Local Health District, Sydney, Australia

**Keywords:** integrated care, people-centred care, universal health coverage, healthcare research, health systems

## Abstract

**Introduction:**

The World Health Organisation (WHO) accepted the Integrated People-centred Health Services (IPCHS) framework in 2016 as an essential component for achieving universal health coverage in fragmented health systems. We aimed to examine the empirical applications of the WHO IPCHS framework to guide its use in strengthening health-service research.

**Methods:**

Academic databases and the IPCHS website were searched for relevant articles published between 2016 and July 2023. Two reviewers independently screened and extracted data on the study design, setting, IPCHS framework components, and facilitators and barriers to implementing the IPCHS strategies. Descriptive and content analyses were conducted.

**Results:**

Six studies were identified using the IPCHS framework. Studies have examined a combination of the five IPCHS strategies. All studies reported building strong primary care-based systems and coordinating care for individuals. Continued relationships and trust, co-production of health programmes, diversity of health care team, and technology were major facilitators, while low health literacy, lack of primary setting capacity and healthcare workforce were principal barriers to IPCHS implementation.

**Conclusion:**

This scoping review offers an overview of IPCHS strategies employed in healthcare research. Generally, the IPCHS framework remains underutilised in primary research. These results offer guidance for future research to support effective healthcare delivery.

## Introduction

Non-communicable diseases (NCDs) are responsible for more than 70% of deaths and disability, and this burden is increasing globally [1]. This growing trend presents significant challenges for health systems, including resource allocation, funding distribution, and the ability to adequately address the needs of patients and the public. To address this, Sustainable Development Goals (SDGs) target 3.4 aims to reduce mortality from NCDs by one-third by 2030 [2]. A renewed focus on service delivery through an integrated and people-centred lens is critical to achieving this, particularly for reaching underserved and marginalised populations to ensure that no one is left behind [3].

The WHO framework on integrated people-centred health services (IPCHS) was developed in consultation and endorsed with strong support from member states at the World Health Assembly in May 2016 [4]. This framework brings a paradigm shift away from disease-centred health systems and institutions towards a more people-centred and integrated approach to healthcare through five interdependent strategies: a) Engaging and empowering people and communities; b) Coordinating services within and across sectors; c) Reorienting the model of care; d) Strengthening governance and accountability; and e) Creating an enabling environment [4]. The WHO also suggested a list of strategic approaches, potential policy options, and interventions to support the attainment of each of the five strategies. The IPCHS framework encourages collaboration and integration across departments, organisations, healthcare institutions, providers and users. It assists governments, development partners, and communities in developing plans and prioritising system reform efforts, regardless of the country’s context or developmental stage. It aids service providers and system leaders in organising, administering, and providing care to better meet individual requirements [5].

Since its introduction in 2016, the IPCHS framework has gained substantial recognition and dissemination across the global health community [5]. Despite the widespread acknowledgement of its principles and the potential it holds for transforming health services, there remains a significant gap in systematic evidence concerning the application of the IPCHS framework within healthcare research. Specifically, the extent to which the IPCHS has been employed as a theoretical foundation to guide research initiatives across diverse healthcare contexts is not well-documented. This lack of comprehensive understanding underscores the need for a scoping review aimed at mapping out the existing research landscape related to the IPCHS framework. By identifying how the framework has been utilised in healthcare research, the strategies and interventions it has informed, and the barriers and facilitators to its implementation, this review seeks to illuminate the current state of IPCHS-inspired research and to identify areas where further investigation is necessary to leverage its full potential for health system strengthening and improvement of healthcare delivery. Specifically, this review aims to address the following focused research questions:

1. How has the IPCHS framework been used in healthcare research?
2. How have specific strategies and interventions outlined in the IPCHS framework been implemented and utilised in research settings?
3. What factors have been identified as barriers or facilitators in the research application of the IPCHS framework, and how can these insights inform strategies to enhance its future research utilisation?

## Methods

A scoping review is deemed most appropriate for capturing the breadth of existing evidence on the WHO IPCHS framework and for outlining its scope in a broad or comprehensive manner. This scoping review was guided by Arksey and O’Malley scoping review methodological framework [6], and reported in accordance with the Preferred Reporting Items for Systematic Reviews and Meta-Analysis extension for Scoping Reviews (PRISMA-ScR) [7].

### Search strategy

Relevant articles were identified by searching academic databases and the WHO IPCHS website. The search of academic databases was conducted in MEDLINE, EMBASE, Global Health, PsychInfo, and Cochrane, using people-centred, patient-centred, person-centred, integrated care, and integrated service as key terms. Search strategies are available in the Supplementary file. To supplement the search from the academic database, we also searched the official WHO IPCHS framework website, which is a platform that serves as a global network for sharing knowledge, practices and facilitating knowledge exchange to transform health services into a more integrated and people-centred approach [8].

### Inclusion and exclusion criteria

Studies were eligible for inclusion if they reported the use of the WHO IPCHS framework and were conducted from 2016 (when the initial report introducing the IPCHS was released) to July 2023. Articles were excluded if they were a) editorials, commentary, or reviews; b) referenced the IPCHS in the introduction or discussion to acknowledge the complexity of the health system but neither applied nor intended to apply the framework; c) not in the English language; and d) abstracts without full text available.

### Study screening and selection

Articles from the two data sources were combined, and duplicate studies were excluded. Two authors (SH, OH, and JY) independently assessed the titles and abstracts of all unique records identified from the academic database and the IPCHS website according to the eligibility criteria. The full texts of potentially eligible studies were then obtained for independent examination by the same authors. The reasons for inclusion or exclusion were recorded, and discrepancies between reviewers were resolved through discussion or with reference to a third author (XY).

### Charting data

Data were extracted into a predefined data charting form in Microsoft Excel for the included studies. The form was piloted using a sample of five randomly selected articles to refine the categories and ensure consistency between reviewers. Two reviewers (XY and SH) charted the data and assessed it for refinement, revising the data charting form in an iterative process. Information on the study design, context, components of the IPCHS framework applied, study outcomes, and the facilitators and barriers of implementing IPCHS strategies were extracted.

### Collating, summarising and reporting the results

The results of the scoping review were summarised in a descriptive, tabular, or format. Using the WHO IPCHS framework and its five strategies as a guide, a narrative synthesis was generated by integration type, demonstrating the implementation of various strategies by countries at various income levels (low-, middle-, and high-income countries), context area, challenges and barriers to using the IPCHS framework, and recommendations for future research. To systematically identify facilitators and barriers in the implementation of the IPCHS framework as reported by the included studies, we employed a content analysis approach. This involved a meticulous extraction process wherein each study was reviewed to identify and code any explicitly mentioned factors that impacted the implementation of IPCHS-related strategies. These factors were then classified as either facilitators or barriers. According to the established scoping review guidelines, the quality appraisal of the included studies was not performed.

## Results

**Figure 1:**
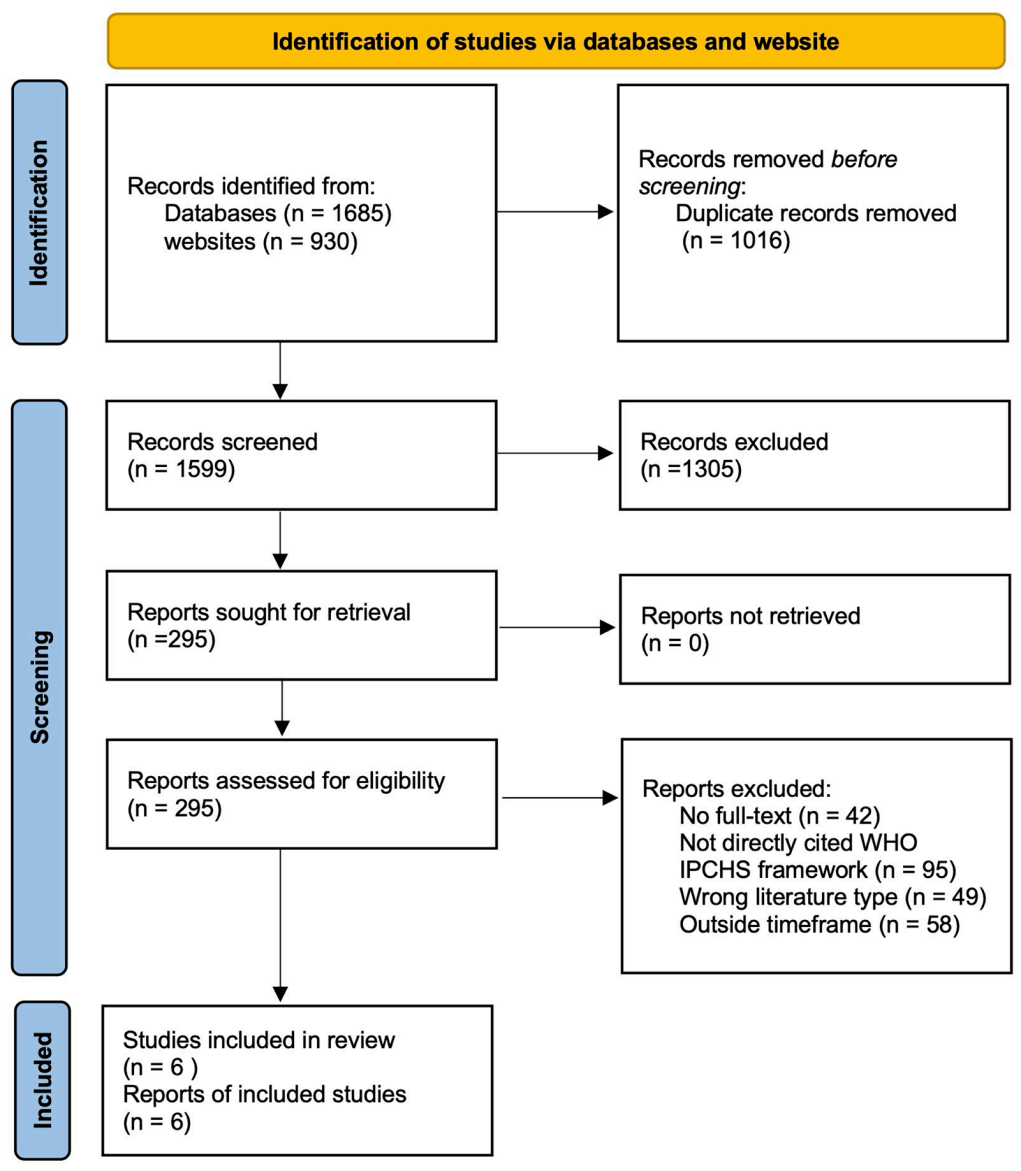
PRIMSA Flowchart.

Following the removal of 1016 duplicates, 2615 unique records were identified through academic databases and the IPCHS website. After screening the titles and abstracts, we retrieved 294 full-text articles for review. Of these articles, 42 were unrelated to the IPCHS framework, 49 had an incorrect article type, and 58 reported studies conducted before 2016. The identification and inclusion of one article in the synthesis was achieved through manual searching. A total of 6 studies were included in the final narrative synthesis that included research articles (n = 3) and protocol papers (n = 3), as shown in the PRISMA flow chart in Figure 1.

### Characteristics of included studies

The WHO IPCHS framework was used in research conducted across four high-income nations, one middle-income nation, and in a multinational context. There were four studies that were qualitative in design, one quantitative study, and one that adopted a mixed methods design. The common qualitative method utilised across the studies was in-depth interviews. The key characteristics of the studies included in this scoping review are presented in Table 1.

**Table 1:** General characteristics of the studies included in this scoping review.


AUTHOR, YEAR	SETTING	STUDY DESIGN	METHODS	SAMPLE SIZE	SAMPLE CHARACTERISTICS	APPLICATION AREAS	OUTCOMES

Verdonck 2023 [9]	WHO Regions	Qualitative study	In-depth interviews	35	Patients with osteoporosis	Osteoporosis care	Patients’ perspectives of patient-centred integrated osteoporosis healthcare

Godinho 2020 [10]	Australia	Mixed methods case study	Documentary analysis and In-depth interviews	NA	Community health alliances	Primary Care	Context, mechanism, facilitators and barriers

Verdonck 2020 [11]	Belgium	Quantitative study	Study protocol	NA	General practitioners and their osteoporosis patients	Primary Care	Patient’s medication possession ratio

Witt 2020 [12]	Australia	Qualitative study	In-depth interviews	26	Community health care provider and health professionals from one tertiary hospital.	Cancer care	Health professionals’ perspectives on communication, continuity and between-service coordination for improving cancer care

Yin 2020 [13]	China	Qualitative study	In-depth interviews	32	Patients with STEMI, cardiologists and nurses from hospitals, emergency department doctors, primary healthcare providers, local health governors, and coordinators at the emergency medical system (EMS)	ST-elevated myocardial infarction	Recommendations for improvement in STEMI treatment

Sullivan-Taylor 2022 [14]	Canada	Qualitative study	In-depth interviews	80	Policy makers, health system decision-makers, Indigenous leaders, providers, patients, caregivers, and academics. (age 65 and over) and those with rare, low-prevalence, and complex diseases.	Theoretical study	Developing IPCHS standards for integrative care. The contents of IPCHS framework


### The application of IPCHS strategies

Studies adopted a combination of strategies. The IPCHS strategies and related sub-strategies used in the included studies are summarised in Table 2. Empowering and engaging communities was a prominent strategy cited in five studies while building strong primary care-based systems, coordinating care for individuals, and coordinating health programmes and providers were cited in all six studies. The most commonly reported interventions include improving awareness and education, enhancing accessibility and coordination of care, using technology, building trust and respect, training and capacity building, improving policy and governance, providing free and accessible healthcare services, and employing treatment guidelines and patient outcome monitoring.

**Table 2:** Overview of studies that make reference to the sub-strategies.


IPCHS STRATEGY	IPCHS SUB-STRATEGY	NUMBER OF STUDIES

1. Engaging and empowering people and communities	1.1 Empowering and engaging individuals and families.	4

	1.2 Empowering and engaging communities.	5

	1.3 Empowering and engaging informal carers.	0

	1.4 Reaching the underserved and marginalised.	2

2. Strengthening governance and accountability;	2.1 Bolstering participatory governance.	3

	2.2 Enhancing mutual accountability.	3

3. Reorienting the model of care;	3.1 Defining service priorities based on life course needs.	3

	3.2 Revaluing promotion, prevention and public health.	2

	3.3 Building strong primary care-based systems.	6

	3.4 Shifting towards more outpatient and ambulatory care.	2

	3.5 Innovating and incorporating new technologies	3

4. Coordinating services within and across sectors;	4.1 Coordinating care for individuals.	6

	4.2 Coordinating health programmes and providers.	5

	4.3 Coordinating across sectors	3

5. Creating an enabling environment.	5.1 Strengthening leadership and management for change.	4

	5.2 Strengthening information systems and knowledge management.	4

	5.3 Striving for quality improvement and safety	3

	5.4 Reorienting the health workforce	3

	5.5 Aligning regulatory frameworks	0

	5.6 Improving funding and reforming payment systems.	2


#### Strategy 1: Empowering and engaging people and communities

Four studies employed empowering and engaging individuals and families. Health awareness campaigns and shared decision-making were emphasised [9,13,14]. For example, in China, where lack of knowledge about STEMI was identified as a patient-level factor of treatment delay, increasing public awareness of ST-segment elevation myocardial infarction symptoms was not only about disseminating information but also aimed at empowering individuals to make timely and informed decisions about seeking care [13]. The integration of digital health with the co-production of health was noted as having the potential to encourage citizen engagement [10]. In essence, the focus was on equipping individuals with the trust, knowledge, and tools required to actively participate and manage their health conditions [9,11]. Empowering and engaging communities was reported by five studies as another prominent sub-strategy. Within this approach, interventions have focused on improving community outreach initiatives and raising public awareness. Health services were provided at township and county levels while addressing particular health issues in nearby communities [13]. Moreover, the establishment of Community Health Alliances (CHAs) illustrated a structured approach to community-level health challenges by fostering a localised support network [10]. Public review and targeted consultation through interviews were conducted to fulfil public needs [14]. Furthermore, one study mentioned that reaching underserved and marginalised populations can provide equitable access to high-quality healthcare services and address the social determinants of health engagement [14]. This encompassed populations residing in geographically disadvantaged areas, indigenous populations, and those with greater degrees of deprivation [12,14].

#### Strategy 2: Strengthening governance and accountability

Strengthening participatory governance was achieved by bolstering participatory governance and enhancing mutual accountability. One study outlines the policy context and the process taken to develop the IPCHS standard, which entailed the constitution of a committee that ensured equitable inclusion of stakeholders, providers, and carers [14]. Case studies in Australia utilise digital health and citizen engagement to deliver integrated people-centred health services (IPCHS) showed that enhancing mutual accountability involved fostering collaborative partnerships among the local health district, primary health network, and local councils, thereby reinforcing accountability within the healthcare system [10]. Furthermore, there was accountability for both system partners and policymakers and the integration of macro and micro directives facilitated the establishment of a coherent governance framework with clearly defined responsibilities [14]. Transparency and accountability have been examined through the incorporation of patient-reported outcome measures, physician outcomes, and patient-reported experience measures. This assessment enabled the identification of areas for improvement and the evaluation of care providers’ performance [11].

#### Strategy 3: Reorienting the model of care

Defining service priorities based on life course needs was mentioned in three studies, emphasising a continuum of care that adapts to changing health requirements. Strengthening primary care-based systems was raised in all six studies. This foundational strategy involves enhancing the capacity of primary healthcare settings to act as the first point of contact. In addition, four studies placed an emphasis on training and capacity building to improve the skills and knowledge of professionals and primary healthcare providers in emergency departments, outpatient centres, and primary healthcare settings [9,11,12,13]. Shifting towards more outpatient and ambulatory care is implemented through many innovative processes that combine with new technologies to expand access to care and bridge gaps in service provision [12]. Innovating and incorporating new technologies was mentioned in three studies [10,11,12]. The use of telemedicine was observed in patient follow-up, making it more convenient and accessible and contributing to a shift towards a robust outpatient model [11]. Support for health and social services has been managed and organised across sectors and organisational boundaries, demonstrating a commitment to an integrated, holistic approach to care provision [12]. Innovating and incorporating new technologies was commonly seen where there is a more active approach in using technology in healthcare delivery and patient communication. Electronic Medical Records were used to facilitate shared patient records and care plans for streamlined communication, and coordinated care received increased attention [12]. In addition to ensuring continuity of care through follow-ups, telehealth has prominent applications in essential services such as chemotherapy [12] and in ensuring continuity of care through follow-ups [11].

#### Strategy 4: Coordinating services within and across sectors

All included studies applied coordinating care for individuals to ensure the systemic integration of health services and improve patient outcomes. In the situation of STEMI treatment delay, there was improved emergency medical services coordination, centralised dispatching of ambulances, and better referral and counter-referral services [13]. Implementation of CHAs also embodies this strategy whereby the focus is on delivering coordinated care at a community level [10]. Coordinating health programmes and providers was implemented where coordination is valued and aligned not only among healthcare providers but also among pharmacists, family physicians, physiotherapists, and nurses [11] but also among different health system decision-makers and stakeholders within a broader public health context [14]. Protocol-driven pathways were used to integrate structure and systematised processes into the overall healthcare system [13]. The coordination of primary and tertiary services in cancer care demonstrated further streamlined pathways at various care levels [12]. Cross-sector collaboration was also evident, with the local health district, primary health network, and local councils [10].

#### Strategy 5: Creating an enabling environment

Four studies changed the way care was delivered using a notable strategy of strengthening leadership and management for change. This involved leaders who were not only committed to the vision of people-centred care but also adept at navigating the complexities of healthcare systems to implement this vision effectively. Furthermore, four studies also implemented the strategy of strengthening information systems and knowledge management by leveraging technology. These studies demonstrated how improved information exchange and communication can streamline clinical processes, reduce errors, and enhance the efficiency of care delivery. The adoption of electronic medical records (EMRs) and telehealth technologies facilitated seamless coordination between different healthcare providers, enabling a more integrated approach to patient care [12,13]. The pursuit of quality improvement and safety was evident where supplementary tools were created to implement and monitor progress [14]. Noteworthy considerations in cancer care included follow-up care and ensuring that healthcare providers possessed adequate cultural and clinical knowledge of indigenous healthcare delivery [12]. Illustrative practices included the development of treatment guidelines and the monitoring of patient outcomes to ensure high-quality and safe care provision [11]. Reorienting the health workforce was seen in three studies through the integration of local knowledge and expertise. Notably, the implementation of targeted group training has been instrumental in enhancing the capacity of healthcare professionals, equipping them with the requisite skills to provide culturally competent care [12,13]. This was achieved through the commitment of staff to acquire knowledge on the cultural and clinical aspects of healthcare delivery for indigenous populations [12]. The provision of health workforce training through personal and postgraduate education was essential for maintaining a continuous process of learning and adaptation [11]. Improving funding and reforming payment systems was seen in two studies where financial intervention was employed to reduce out-of-pocket costs for patients [13]. However, there were concerns related to the affordability of treatments, especially for those with private healthcare [9].

### Barriers and facilitators in the implementation of the IPCHS framework

This study identified various barriers and facilitators to implementing IPCHS strategies, as summarised in Table 3. Patients’ positive attitudes, continued relationships, trust in health providers [12], respecting patients’ perspectives in clinical decision-making [13,14], and healthcare team diversity [12] were identified to facilitate the implementation of engaging and empowering people and communities. Barriers identified hindering the reorientation of the care included a lack of workforce [13] and a heavy workload [12]. The adoption of a holistic approach and the provision of multidisciplinary care have emerged as facilitators of this approach [9]. Technology emerged as a significant facilitator for reorienting the model of care [12]. It provided valuable assistance for staff in primary care settings, enabling efficient management of patient information, streamlined communication, and improved coordination of care [10,12,13]. Technology also offered flexibility for health providers in high-level hospitals, allowing them to access patient data remotely, consult with colleagues, and make informed decisions, ultimately enhancing the quality and efficiency of care delivery [12]. Communication gaps, lack of coordination and integration among different components of the health care system, lack of standardised electronic platforms, and inflexibility in care provision were challenges for coordinating services within and across sectors [9,12,13]. On the contrary, facilitators encompassed timely information exchange, collaborative approaches to patient care, and streamlined processes for efficient information sharing [12,13].

**Table 3:** Identified facilitators and barriers for implementing the IPCHS strategies.


STRATEGIES	FACILITATORS	BARRIERS

**Engaging and empowering people and communities**	Patient advocacy and involvement [9]; Continued relationships and trust with providers [9,12]; Value competencies of staff members [9];	Lack of awareness [9]; Lack of knowledge [9,13]; Paternalistic approach and poor therapeutic alliances [9]; Patient concerns belittled [9]; Lack of shared decision making [9]; Lack of a holistic approach [9];

**Strengthening governance and accountability**		Lack of policy support [13];

**Reorienting the model of care**	Technology, such as m-health, telemedicine [11,12]; Integrated care models with multidisciplinary care [9]; More holistic approach [9];	Lack of training for primary care providers [13]; Heavy workload of hospital staff [12]; Limited capacity of professionals in health system [13]; Inadequate staff knowledge [12]; Inequities in care [9]; Long waiting times for investigations [9]; Absence of primary care gatekeeping secondary care [9]; Limited awareness and prevention [9]; Late promotion of health [9];

**Coordinating services within and across sectors**	Linking promotive and preventive healthcare to primary care [9]; Timely communication and information exchange [12,13]; Specialised clinics [9]; Personalised care [9];	Lack of coordination between hospitals at different levels [9,12]; Siloed care fragmentation [9,12]; Ineffective administration [12]; Delayed communication and information exchange on patients and condition [12]; Financial barriers to patient referrals in resource-constrained areas [12]; Lack of care pathways [9]; Lack of alternative treatments acknowledgement [9];

**Creating an enabling environment**	Cultural appreciation [12]; Proactive approach to patient care [12];	Lack of medical equipment in primary settings [13]; Lack of system processes and streamlined services [12]; Financial barriers to care [9,13];


## Discussion

This study, since the inception of the WHO IPCHS framework, outlined an overview of the studies that used the WHO IPCHS framework for research in various contexts along with its facilitators and barriers. The direct utilisation of the WHO IPCHS framework in empirical research has not reached the anticipated level of widespread adoption. Nonetheless, its outcomes are useful in guiding proponents of integrated care in people-centred contexts.

### How has The IPCHS framework been used in healthcare research?

With the growing recognition of patients not just as care recipients but as central participators within a health system, the significance of person-centred care has never been more crucial [15]. It has been used as a guiding tool to help reform healthcare practices and systems [9,13,14], and has been proven effective in identifying gaps in the existing health system and subsequently used to inform strategies and recommendations [9]. It also offered a systematic lens for organising data and interpreting, as well as for the conceptualisation of the study design and objectives [11,12]. However, the WHO IPCHS framework has documented a somewhat gradual integration into empirical research despite its recognised potential. This phenomenon may be partly attributed to the limited awareness and familiarity among researchers and practitioners and the availability of alternative models [15,16]. The complexity of the framework itself adds to its gradual integration into research due to its extensive scope and holistic approach, intersectoral collaboration, varied array of stakeholder participation, comprehensive multi-dimensional metrics required for measurement and evaluation, and degrees of transformational administration. Resource constraints and research traditions within specific fields can further contribute to its limited direct utilisation [17]. There is an imperative need for concerted efforts in dissemination and promotion. These include education and training initiatives, advocacy for explicit citations and applications in leading research journals, fostering collaboration among healthcare institutions, and showcasing successful case studies. As the healthcare landscape continues to evolve, promoting the adoption of the IPCHS framework remains pivotal in shaping the future of patient-centred care and healthcare research.

### How have specific strategies and interventions outlined in the IPCHS framework been implemented and utilised in research settings?

The implementation of the WHO IPCHS framework strategies across diverse healthcare contexts has yielded varying outcomes, encompassing empowering individuals and communities, enhancing governance and accountability, reorienting care models, coordinating services, and creating enabling environments. These strategies, while generally adopted, exhibit variations in the utilisation of their sub-strategies. We observed the strategy of empowering and engaging informal carers was absent from the included studies. This oversight is intriguing, especially in light of evidence demonstrating the efficacy of interventions such as occupational therapy [18], and psychoeducation [19] in enhancing carer outcomes and the overall quality of life for those under care in enhancing carer outcomes and the overall quality of life for those under care. This aligns with the IPCHS framework’s emphasis on supporting and educating caregivers, suggesting a missed opportunity in the literature to explore how such strategies can be integrated into health services. The underrepresentation of this domain may reflect a broader trend in health service research, where the indispensable role of informal carers is often under-recognised. Equally, the strategy of aligning regulatory frameworks was not referenced in any reviewed studies. Regulatory frameworks are fundamental in shaping health service delivery, yet their complex and often bureaucratic nature may pose challenges for empirical investigation. Despite these challenges, research outside our review has underscored the value of transparent, supportive regulatory practices in elevating healthcare standards. This suggests a critical gap in the literature and a pressing need for studies that delve into how regulatory frameworks can be aligned with people-centred health services to enhance care delivery. Further research is essential to fully assess the impact of these strategic approaches and practice interventions, particularly in terms of their effectiveness in improving healthcare outcomes and addressing disparities.

### What factors have been identified as barriers or facilitators in the research application of the IPCHS framework, and how can these insights inform strategies to enhance its future research utilisation

The examination of facilitators and barriers in the research application of the IPCHS framework underscores critical elements in the transformation of healthcare towards an integrated, patient-centred model. Firstly, while active patient engagement fosters trust, when we look at the barriers for the strategy of empowering and engaging communities, it was perceived that there was a lack of underlying knowledge and awareness. Active patient engagement fosters trust, yet challenges are linked to a paternalistic approach. Fortunately, facilitators included the mechanisms of human behaviour of trust, relationship and appreciation, which was facilitated by telehealth and also timely communications. Therefore, an implication for research utilisation is understanding the context and needs of the patient, carer, and families is key, and for the need to have co-design models of care and to have communication platforms that encourage trust and respect throughout the process from the beginning to end [20].

For the other IPCHS strategies, staffing, financial and human resource constraints, including medical equipment, were mentioned as barriers to the implementation of the strategies. This highlights the interconnectedness and dependency of these strategies in our complex healthcare system and the need for an enabling environment. For instance, the absence of robust government policy support constitutes a formidable obstacle to reinforcing governance and accountability, which is paramount for a comprehensive systemic transformation. Policymakers should prioritise policy dialogue and the efficient development and implementation of the IPCHS framework into the healthcare system. Furthermore, technology, multidisciplinary teams, and holistic care bolster the reorientation of the care model; however, disparities, delayed health promotion, and training deficits persist. Addressing the underlying causes of health inequalities and focusing on the social determinants of health may yield further insight. Effective communication enhances coordination services within and across sectors, whereas care fragmentation and administrative inefficiencies continue to impede coordination, underscoring the imperative for improved communication and coordination, as endorsed by the IPCHS framework. Creating an enabling environment benefits from cultural sensitivity but confronts financial and procedural barriers. Streamlining administrative processes by adopting unified electronic health record systems and implementing standardised care pathways can enhance coordination and reduce fragmentation [21]. To address financial barriers, innovative funding models and targeted policy interventions are required. At the individual level, obstacles related to finances can be facilitated through social aid programs and initiatives focused on cultivating good physical and mental welfare. This approach supports individuals in accessing necessary healthcare services without the burden of financial strain. At the organisational level, healthcare systems are obligated to guarantee that medical institutions provide accessible financial amenities. This includes promoting transparency in billing and pricing and ensuring fair access to available resources, thereby reducing disparities in healthcare access and affordability [22].

### Limitations of this study

This study is the first scoping review to examine academic literature that uses the IPCHS framework systematically. A limitation of this study is the lack of an outcome assessment, which consequently restricts our capacity to determine the effectiveness of IPCHS strategies definitively. In addition, it should be noted that one study was discovered through manual searching and was not initially identified in the database, as it may not have been indexed and fit within our strict inclusion criteria. Therefore, additional articles could have been overlooked. In addition, a comprehensive search of grey literature was not conducted. As a result, non-academic literature, including policy documents and implementation reports, was not considered. This limitation suggests that while our review offers a focused overview of the IPCHS framework’s academic applications, it may not fully capture the broader context of its integration into health systems and policies. Future research could benefit from an expanded search strategy that encompasses both academic and grey literature to provide a more holistic view of the IPCHS framework’s adoption and impact. One final limitation of this study is the lack of an outcome assessment, which consequently restricts our capacity to determine the effectiveness of IPCHS strategies definitively.

## Conclusion

The WHO IPCHS framework has emerged as a valuable asset in the arsenal of healthcare research methodologies. Its multifaceted utility encompasses guiding healthcare reform efforts, identifying system deficiencies, structuring research endeavours, and fostering innovation through collaborative digital approaches. While its direct utilisation may be limited, harnessing the IPCHS framework’s insights represents a crucial step towards strengthening health-service research and ultimately improving healthcare outcomes. This scoping review highlights the imperative for broader dissemination and application of the IPCHS framework among healthcare researchers and practitioners. Moving forward, there is a critical need for empirical studies focused on assessing the impact of implementing people-centred health services in specific patient populations. Such research would not only contribute to a deeper understanding of the framework’s value but also aid in tailoring health services to meet the unique needs of diverse patient groups.

## Additional File

The additional file for this article can be found as follows:

10.5334/ijic.7754.s1 Supplementary file. Data source.
