# Supplementary material for: Examining the Use and Application of the WHO Integrated People-Centred Health Services Framework in Research Globally – a Systematic Scoping Review
Source: Int J Integr Care. 2024 Apr 25;24(2):9. doi: 10.5334/ijic.7754 (PMC11049668; doi:10.5334/ijic.7754)
Supplement: Supplementary file. — Data source. [file ijic-24-2-7754-s1.pdf]

Data source:

A. WHO IPCHS website: https://www.integratedcare4people.org/publications/
Filter: Type=scientific publication

B. Literature search:
Database: MEDLINE, EMBASE, Global Health, PsychInfo and Cochrane via Ovid
Search strategy:

1. people-cent$.ti,ab.
2. patient-cent$.ti,ab.
3. person-cent$.ti,ab.
4. integrat$ adj care
5. integrat$ adj service?
6. 1 or 2 or 3
7. 4 or 5
8. 6&7
9. limit 8 to yr="2015 - Current"
10. **remove duplicates from 9**
